# Visit complexity, diagnostic uncertainty, and antibiotic prescribing for acute cough in primary care: a retrospective study

**DOI:** 10.1186/1471-2296-14-120

**Published:** 2013-08-19

**Authors:** Lauren E Whaley, Alexandra C Businger, Patrick P Dempsey, Jeffrey A Linder

**Affiliations:** 1 Division of General Medicine and Primary Care, Brigham and Women’s Hospital, Boston, MA, USA; 2 Harvard Medical School, Boston, MA, USA

**Keywords:** Antibacterial agents, Respiratory tract infections, Cough, Physicians, Primary care, Decision making, Diagnosis

## Abstract

**Background:**

Guidelines and performance measures recommend avoiding antibiotics for acute cough/acute bronchitis and presume visits are straightforward with simple diagnostic decision-making. We evaluated clinician-assigned diagnoses, diagnostic uncertainty, and antibiotic prescribing for acute cough visits in primary care.

**Methods:**

We conducted a retrospective analysis of acute cough visits – cough lasting ≤21 days in adults 18–64 years old without chronic lung disease – in a primary care practice from March 2011 through June 2012.

**Results:**

Of 56,301 visits, 962 (2%) were for acute cough. Clinicians diagnosed patients with 1, 2, or ≥ 3 cough-related diagnoses in 54%, 35%, and 11% of visits, respectively. The most common principal diagnoses were upper respiratory infection (46%), sinusitis (10%), acute bronchitis (9%), and pneumonia (8%). Clinicians prescribed antibiotics in 22% of all visits: 65% of visits with antibiotic-appropriate diagnoses and 4% of visits with non-antibiotic-appropriate diagnoses. Clinicians expressed diagnostic uncertainty in 16% of all visits: 43% of visits with antibiotic-appropriate diagnoses and 5% of visits with non-antibiotic-appropriate diagnoses. Clinicians expressed uncertainty more often when prescribing antibiotics than when not prescribing antibiotics (30% vs. 12%; p < 0.001). As the number of visit diagnoses increased from 1 to 2 to ≥ 3, clinicians were more likely to express diagnostic uncertainty (5%, 25%, 40%, respectively; p < 0.001) and prescribe antibiotics (16%, 25%, 41%, respectively; p < 0.001).

**Conclusions:**

Acute cough may be more complex and have more diagnostic uncertainty than guidelines and performance measures presume. Efforts to reduce antibiotic prescribing for acute cough should address diagnostic complexity and uncertainty that clinicians face.

## Background

Guidelines and performance measures recommend avoiding routine antibiotic prescribing for patients with acute cough/acute bronchitis [1]–[3]. These guidelines and performance measures are based on randomized controlled trials and meta-analyses showing that antibiotics are not beneficial, and may even be harmful, for adults with acute cough [4,5]. Despite the evidence and recommendations, physicians in the United States continue to prescribe antibiotics to adults with acute bronchitis at 65% of visits [6].

Part of the gap between recommendations and actual practice may be that guidelines and performance measures presume visits are straightforward with simple diagnostic decision-making, when, in fact, they are more complex. The American College of Physicians/Centers for Disease Control and Prevention guideline, “Principles of appropriate antibiotic use for treatment of acute bronchitis in adults” does not apply to patients with cardiopulmonary disease and focuses on excluding the diagnosis of pneumonia, but does not mention the possibility of other concomitant, cough-related diagnoses [1,7]. The Healthcare Effectiveness Data and Information Set (HEDIS) performance measure, “Avoidance of Antibiotic Treatment in Adults With Acute Bronchitis,” includes a single diagnosis code, and has a list of exclusionary ICD-9 codes for chronic lung disease, immunosuppression, and malignancy, but does not mention diagnostic uncertainty [3].

We conducted a retrospective chart review to measure the antibiotic prescribing rate and measure the prevalence of diagnostic complexity and diagnostic uncertainty that clinicians face when treating patients with acute cough.

## Methods

### Overview

We performed a retrospective analysis to identify acute cough visits to a single primary care practice. We included visits by patients aged 18 to 64 years old, with a cough lasting up to 21 days, and without chronic lung disease. This analysis was part of an evaluation to inform an intervention targeted at reducing antibiotic prescribing for acute cough/acute bronchitis throughout our Practice Based Research Network.

### Setting

The Phyllis Jen Center for Primary Care is a teaching practice in the Brigham and Women’s Primary Care Practice Based Research Network. The practice has over 40 faculty physicians and 80 internal medicine residents with continuity clinics each academic year. About 40 additional internal medicine residents rotate through the practice for urgent care visits. There are also 3 Nurse Practitioners, 2 Registered Nurses, 2 Pharmacy Technicians, 2 Pharmacists, and 1 Social Worker. The practice serves a socioeconomically, racially, and ethnically diverse patient population.

The practice uses the Longitudinal Medical Record as the official electronic health record (EHR) [8]. The EHR was internally developed by Partners HealthCare, of which Brigham and Women’s Hospital is a founding member. The EHR includes primary care and subspecialty notes, problem lists, medication lists, coded allergies, and laboratory test and radiographic study results. The EHR has clinical decision support, but not pertaining to the antibiotic treatment of acute respiratory infections.

### Data sources

Using the EHR schedule, we identified all visits made to the practice between March 1, 2011 and June 30, 2012. We included only patients aged 18 to 64 years old to match the age range of the HEDIS performance measure. We excluded patients who had made a visit to the practice in the prior 30 days, and patients with chronic lung disease on their problem list. Our list of chronic lung disease diagnoses included, but was not limited to, asthma, asthmatic bronchitis, chronic obstructive pulmonary disease, interstitial lung disease, and chronic bronchitis.

### Data extraction

For patient visits that met our inclusion criteria, we reviewed the EHR note to identify patients with acute cough. We verified that patients met a guideline-consistent definition of acute cough/acute bronchitis: a cough lasting 21 or fewer days in a patient age 18 to 64 years old without chronic lung disease or immunosuppression [1,2]. To be included in our analysis, patients did not have to have a chief complaint of cough documented in the visit note. If the treating clinician did not mention the duration of cough, study staff contacted the clinician to ascertain whether the cough was acute (≤ 21 days). We excluded visits at which the clinician did not address acute cough in their treatment plan.

To assess inclusion and exclusion criteria and to record information about acute cough visits, we imported and manually extracted acute cough visit data into a study-specific database (Microsoft Access 2003). We imported data including date, provider name, patient identification number, age, sex, race/ethnicity (from registration data) and vital signs. We extracted clinical documentation data about the patient’s chief complaint or reason for visit, symptoms, physical exam findings, diagnoses, and treatments. For symptoms, we also noted whether the following eight symptoms were present, not present, or not mentioned: sore throat, phlegm/sputum, shortness of breath, headache, fevers, nasal symptoms, myalgias, and chest pain.

For the physical exam, we extracted information on the normality, abnormality, or lack of mention about general appearance, tympanic membranes, external auditory canals, oropharynx, lymphadenopathy, lung sounds, or wheezing. Finally, we recorded the presence or absence of a chest x-ray, and whether the results were normal or abnormal.

We extracted up to three clinician-assigned diagnoses as documented in the assessment and plan section of the visit note. For each visit, we assigned one of 16 common diagnoses associated with an acute cough. We considered sinusitis, pneumonia, streptococcal pharyngitis, otitis media, bacterial infection, and pertussis “antibiotic-appropriate diagnoses”. We use the term “antibiotic-appropriate diagnoses” for diagnoses for which some patients may require antibiotics according to guidelines (e.g., sinusitis with severe symptoms) even though the majority of patients with “antibiotic-appropriate diagnoses” may not require antibiotics. We considered upper respiratory infection (URI), acute bronchitis, viral syndrome, post-nasal drip, non-streptococcal pharyngitis, allergies, reactive airway disease, gastroesophageal reflux disease, conjunctivitis, and influenza “non-antibiotic-appropriate diagnoses”. For diagnoses not in one of these 16 categories, we classified them as “other” and considered “other” a non-antibiotic-appropriate diagnosis. Even though some of these diagnoses might more rightly be considered symptoms (e.g., post-nasal drip), these were the diagnoses assigned by the treating clinician.

We noted when the clinician expressed uncertainty when assigning the diagnosis, such as when providers used words like “maybe,” “unclear,” or used question marks in association with the diagnosis. As examples, we considered notes to express diagnostic uncertainty if they included text such as the patient “had a cough and sore throat with unclear etiology” or “presenting with ?acute bronchitis”. We also considered clinicians’ weighing of multiple possible diagnoses an expression of uncertainty (e.g., “pneumonia versus acute bronchitis” or “URI or possible underlying sinusitis”). We did not consider clinicians documenting two distinct diagnoses an expression of uncertainty (e.g. “pneumonia with acute bronchitis” or “URI with sinusitis”).

We collected and documented up to four prescribed medications as recorded in the electronic health record, giving preference to antibiotics and cough-related treatments. We did not abstract information about non-cough-related diagnoses or associated antibiotics (e.g. amoxicillin given to a patient with a urinary tract infection). We did not distinguish between immediate antibiotic prescribing and “delayed antibiotic prescribing”. Delayed antibiotic prescribing was very rare and guidelines and performance measures make no distinction or allowance for delayed antibiotic prescribing [1-3].

We extracted information about whether patients made any follow-up visits to an affiliated site or received any antibiotic prescriptions within the 30 days following their initial visit. For each follow-up visit made, we noted where the care was delivered (emergency department, primary care practice, specialist, etc.), the subject of the visit note, and whether the visit was related to acute bronchitis and/or antibiotic prescribing. If the follow-up visit was cough-related, we extracted up to three diagnoses, whether the clinicians expressed diagnostic uncertainty, and/or prescribed antibiotics.

### Data and statistical analysis

For each visit, we assigned a principal diagnosis. We prioritized the principal diagnosis by giving preference to the most antibiotic-appropriate diagnosis mentioned in a given visit in the following order: pneumonia, sinusitis, streptococcal pharyngitis, otitis media, pertussis, non-streptococcal pharyngitis, acute bronchitis, upper respiratory infection, post nasal drip, allergies, and other. For example, if a visit was given both URI and pneumonia diagnoses, the visit was assigned a principal diagnosis of pneumonia.

We used standard descriptive statistics to describe and compare baseline characteristics of physicians and patients. We compared continuous variables using Student’s t test and categorical variables using the × [2] test. We used SAS version 9.2 (SAS Institute Inc, Cary, NC). We considered p-values < 0.05 significant.

The Partners HealthCare Human Research Committee approved the study protocol.

## Results

### Visit flow

**Figure 1:**
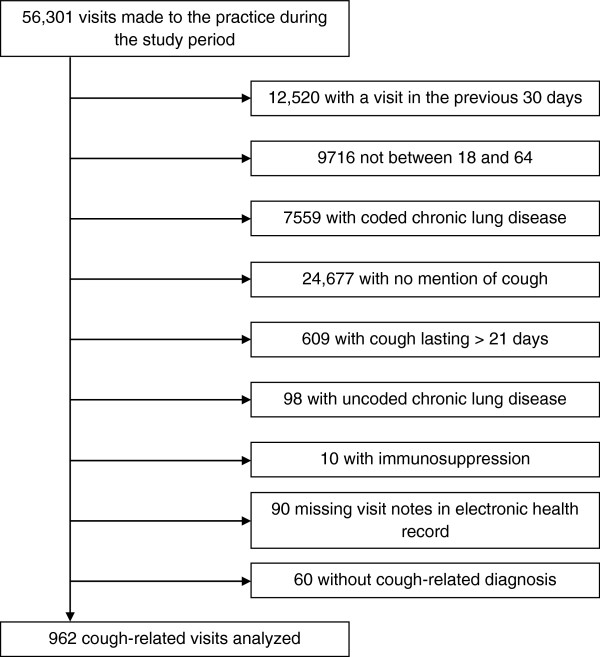
Visit flow.

During the study period, there were 56,301 visits to the practice. We excluded 31,017 (54%) visits due to age, the presence of chronic lung disease, and having had a visit in the previous 30 days (Figure 1). We reviewed notes from 26,506 visits made by 11,007 patients. There were 1022 visits that met our definition of acute cough. Of these visits, 60 of the visit notes did not have a cough-related diagnosis in the assessment or plan and were excluded from diagnosis- and medication-related analyses. This left 962 acute cough visits with a cough-related diagnosis in the analysis (2% of all visits), of which 399 (41%) were seen by residents.

### Patient and illness characteristics

Among the 962 acute cough-related visits, there were 944 patients who saw 193 different clinicians: 143 residents, 47 attending physicians, and 3 nurse practitioners. Patients were 74% female, 45% white, and with a mean age of 45 years (Table 1). Residents saw 41% of the visits. The mean duration of cough was 8 days. Of these visits, 500 (52%) had cough as their chief complaint. The most common non-cough symptoms were nasal congestion (58%), sore throat (49%), phlegm (39%), and fever (28%; Table 2). The most common findings on physical examination were oropharyngeal abnormality (26%), lymphadenopathy (14%), tympanic membrane or external auditory canal abnormality (12%), and overall abnormal appearance (12%; e.g., ill-appearing). Chest x-rays were ordered in 11% of visits, of which 31 (30%) had abnormal results.

**Table 1:** **Visit characteristics (N = 962)**^**1**^

**Characteristic**	
	**Mean (SD)**
Age	45 (12)
	**N (%)**
Sex	
Female	710 (74)
Male	252 (26)
Race and Ethnicity	
White, non-Latino	437 (45)
Black, non-Latino	278 (29)
Latino	160 (17)
Asian	53 (6)
Unavailable/Other	34 (4)
Insurance Status	
Private	683 (71)
Medicaid	182 (19)
Medicare	81 (8)
Self-Pay	15 (2)
Co-morbidities	
Diabetes	108 (11)
Cancer	51 (5)
Rheumatologic disease	39 (5)
Mild liver disease	31 (3)
Myocardial infarction	19 (2)
Cerebrovascular disease	17 (2)
Renal disease	15 (2)

^1^ Some groups may not add up to 100% because of rounding.

**Table 2:** Patient characteristics and antibiotic prescribing (N = 962)

**Characteristic**	**Yes**	**No**	**Not mentioned**	**Prescribed antibiotics**
	**N (%)**			**N (%)**
***Symptoms***				
Nasal symptoms	556 (58)	112 (12)	294 (31)	132 (24)
Sore throat	471 (49)	120 (12)	371 (39)	101 (21)
Phlegm/sputum	375 (39)	316 (32)	271 (28)	91 (24)
Fevers	272 (28)	475 (49)	215 (22)	76 (28)
Headache	223 (23)	122 (13)	617 (64)	64 (29)
Myalgias	187 (19)	58 (6)	717 (75)	46 (25)
Shortness of breath	134 (14)	352 (37)	476 (49)	34 (25)
Chest pain	108 (11)	263 (27)	591 (61)	19 (18)
***Physical exam findings***				
Oropharyngeal abnormality	248 (26)	496 (52)	218 (23)	54 (22)
Lymphadenopathy	132 (14)	613 (64)	217 (23)	43 (33)
Tympanic membrane or external auditory canal abnormality	117 (12)	296 (31)	549 (57)	39 (33)
Abnormal appearance	114 (12)	632 (66)	216 (22)	38 (33)
Abnormal lung sounds	106 (11)	755 (78)	101 (10)	46 (43)
Wheezing	100 (10)	394 (41)	468 (49)	27 (27)

### Diagnoses and diagnostic uncertainty

For the 962 acute cough visits, physicians made 1510 cough-related diagnoses, most commonly URI (41%), post-nasal drip (9%), acute bronchitis (8%), and sinusitis (7%). After prioritizing cough-related diagnoses from most antibiotic-appropriate to least, the most common principal cough-related diagnoses were URI (46%), sinusitis (10%), acute bronchitis (9%), and pneumonia (8%; Table 3). There was no difference between residents and non-residents in diagnosing patients with antibiotic-appropriate diagnoses or non-antibiotic-appropriate diagnoses (p = 0.15; Table 3).

**Table 3:** **Principal diagnoses**^**1**^

**Diagnoses**	**Total**	**Residents**	**Non-residents**	**Given antibiotics**	**Diagnostic uncertainty**
**N (%)**					
***Antibiotic-appropriate***					
Sinusitis	99 (10)	28 (7)	71 (13)	77 (78)	19 (19)
Pneumonia	74 (8)	32 (8)	42 (7)	51 (69)	36 (49)
Streptococcal pharyngitis	69 (7)	26 (7)	43 (8)	27 (39)	49 (71)
Otitis media	16 (2)	6 (2)	10 (2)	13 (81)	1 (6)
Bacterial infection^2^	10 (1)	3 (1)	7 (1)	6 (60)	8 (80)
Pertussis	9 (1)	4 (1)	5 (1)	6 (67)	6 (67)
***Subtotal***	277 (29)	99 (25)	178 (32)	180 (65)	119 (43)
***Non-antibiotic-appropriate***					
Upper respiratory infection	445 (46)	192 (48)	253 (45)	14 (3)	12 (3)
Acute bronchitis	90 (9)	34 (9)	56 (10)	13 (14)	4 (4)
Post-nasal drip	33 (3)	15 (4)	18 (3)	1 (3)	3 (9)
Non-streptococcal pharyngitis	33 (3)	18 (5)	15 (3)	0 (0)	5 (15)
Allergies	32 (3)	17 (4)	15 (3)	0 (0)	1 (3)
Reactive airway disease	5 (1)	1 (0)	4 (1)	0 (0)	1 (20)
Other^3^	47 (5)	23 (6)	24 (4)	2 (4)	6 (13)
***Subtotal***	685 (71)	300 (75)	385 (68)	29 (4)	32 (5)
**Total**	**962 (100)**	**399 (100)**	**563 (100)**	**209 (22)**	**151 (16)**

^1^ Total, Resident, and Non-resident percentages are column percents. Given antibiotics and Diagnostic uncertainty are row percents. Percentages may not add up because of rounding.

^2^ “Bacterial infection” was most often written as “bacterial superinfection”.

^3^ “Other” includes diagnoses that were listed 5 times or fewer, or that listed “cough” as a diagnosis. The most common other diagnoses were “cough” (16), influenza (4), and gastroesophageal reflux disease (3).

Clinicians expressed diagnostic uncertainty in 151 visits (16%). Clinicians expressed diagnostic uncertainty more often for antibiotic-appropriate diagnoses (43%) than for non-antibiotic-appropriate diagnoses (5%; p < 0.001). Clinicians were more likely to express diagnostic uncertainty with increasing number of diagnoses: 5% for 1 diagnosis, 25% for 2 diagnoses, and 40% for 3 or more diagnoses (p < 0.0001; Table 4). Residents were not more likely to assign multiple diagnoses (41% versus 49% for non-residents, p = 0.07) or express diagnostic uncertainty (14% versus 17% for non-residents; p = 0.22). Clinicians were more likely to order a chest x-ray when expressing diagnostic uncertainty (21%) than when not expressing diagnostic uncertainty (9%; p < 0.0001).

**Table 4:** Number of cough-related diagnoses, antibiotic prescribing, and diagnostic uncertainty

**Number of cough-related diagnoses**	**Visits**	**Antibiotics prescribed**	**Diagnostic uncertainty**
**N (%)**			
1 diagnosis	519 (54)	82 (16)	24 (5)
2 diagnoses	338 (35)	84 (25)	85 (25)
3+ diagnoses	105 (11)	43 (41)	42 (40)
**Total**	**962 (100)**	**209 (22)**	**151 (16)**

### Medications and antibiotic prescribing

Clinicians listed a total of 1596 prescription and over-the-counter medications in their visit notes. The most common primary medications prescribed were antibiotics (22%), antitussives or expectorants (17%), decongestants (16%), analgesic/antipyretics (8%), and bronchodilators (7%). The most common classes of antibiotics were macrolides (53%), penicillins (28%), and fluoroquinolones (10%). Clinicians prescribed antibiotics in 65% (180/277) of visits with antibiotic-appropriate diagnoses, and 4% (29/685) of visits with non-antibiotic-appropriate diagnoses (Table 3). Of visits in which a chest x-ray was ordered, 46 (45%) received an antibiotic: 65% with abnormal and 37% with normal chest x-ray results.

Clinicians expressed diagnostic uncertainty in 65% of the antibiotic-appropriate visits that did not receive an antibiotic. Non-antibiotic-appropriate diagnoses accounted for 14% (29/209) of antibiotic prescribing.

Residents were less likely to prescribe antibiotics overall (17% versus 25% for non-residents; p < 0.01). Residents did not prescribe antibiotics at different rates for antibiotic-appropriate diagnoses (60% versus 68% for non-residents; p = 0.4) or for non-antibiotic-appropriate diagnoses (3% versus 5% for non-residents; p = 0.07).

As the number of diagnoses increased, clinicians were more likely to prescribe antibiotics: 16% for 1 diagnosis, 25% for 2 diagnoses, 41% for 3 or more diagnoses, respectively (p < 0.001; Table 4). Clinicians expressed uncertainty more often when prescribing antibiotics (30%) than when not prescribing antibiotics (12%; p < 0.001).

### Follow-up visits

Within 30 days, 158 (16%) initial visits resulted in 205 cough-related follow-up visits. These visits were to the same clinic as their initial visit (76%); to a specialist (12%); to an emergency department (10%); or to a different primary care practice (3%). Initial visits with a follow-up visit were more likely to have had an antibiotic-appropriate diagnosis (40%) compared to initial visits without a follow-up visit (27%, p < 0.001). Initial visits with a follow-up visit were also more likely to have had an antibiotic prescription (21%) compared to initial visits without a follow-up visit (15%, p < 0.05). There was no significant difference in diagnostic uncertainty between visits that did (19%) and did not have a follow-up visit (15%, p = 0.25).

When comparing initial visit and follow-up diagnoses, 62% of follow-up visits had a different primary diagnosis than in the original visit note; 18% of visits changed from an antibiotic-appropriate diagnosis to a non-antibiotic-appropriate diagnosis; and 17% changed from non-antibiotic-appropriate to antibiotic-appropriate. Of all initial visits, 76 (8%) patients received an antibiotic prescription within 30 days. There was no difference in antibiotic prescribing at follow-up visits at the study clinic (29%) versus other sites (20%, p = 0.23).

## Discussion

Guidelines and performance measures for antibiotic prescribing for acute cough/acute bronchitis presume that visits are simple and decision-making is straightforward. In this retrospective chart review, 46% of visits had more than one cough-related diagnosis and clinicians expressed diagnostic uncertainty in 16% of visits. Clinicians prescribed antibiotics more commonly for visits with antibiotic-appropriate diagnoses, with an increasing number of cough-related diagnoses, and at which the clinician expressed diagnostic uncertainty.

Clinicians prescribed antibiotics at 65% of visits with antibiotic-appropriate diagnoses and 4% with non-antibiotic-appropriate diagnoses. Although we use the term “antibiotic-appropriate diagnosis”, not all of these visits require antibiotics, like mild sinusitis of short duration [9,10], suspected pertussis for more than 3 weeks (to reduce infectivity) [11], or a provisional diagnosis of streptococcal pharyngitis. Diagnoses for cough and the appropriateness of antibiotic prescribing are often considered in isolation. However, a broader range of diagnoses – antibiotic-appropriate and non-antibiotic-appropriate – are helpful to understand clinicians’ treatment decisions. For example, clinicians can easily avoid the appearance of inappropriate antibiotic prescribing for acute bronchitis, a non-antibiotic-appropriate diagnosis, through diagnosis selection [12].

Clinicians were more likely to prescribe antibiotics at visits with more cough-related diagnoses. Logically, the more diagnoses, the more likely one diagnosis would be antibiotic-appropriate. Beyond the presence of antibiotic-appropriate diagnoses, having more than one cough-related diagnosis may indicate greater patient complexity or an implicit expression of diagnostic uncertainty.

Explicit expressions of diagnostic uncertainty were associated with antibiotic prescribing. In addition, clinicians seemed to express diagnostic uncertainty even more when there was a mismatch between the diagnosis and antibiotic treatment: clinicians expressed diagnostic uncertainty in 43% of antibiotic-appropriate diagnosis visits and 65% of antibiotic-appropriate diagnosis visits at which an antibiotic was *not* prescribed. More directly stated, when there is uncertainty, the treatment is less likely to match the diagnosis. Clinicians were more likely to express diagnostic uncertainty when they gave more than one diagnosis for the visit, and suggest diagnostic ambiguity when listing symptoms such as “cough” as a diagnosis. These associations point to a confluence between diagnostic uncertainty, increasing number of diagnoses, antibiotic-appropriate diagnoses, and antibiotic prescribing.

Although clinicians may assuage their uncertainty with an antibiotic-appropriate diagnosis or an antibiotic prescription, patients do not do better. Initial visits with an antibiotic-appropriate primary diagnosis or with an antibiotic prescription were more likely to have a follow-up visit. Our analysis cannot answer whether these patients would not have had follow-up visits if the patients had been given non-antibiotic-appropriate diagnoses or had not been given antibiotics. A qualitative, European study suggests that clinicians decrease their antibiotic prescribing with an increase in clinical experience and diagnostic confidence [13].

Our analysis has limitations that should be considered. First, the analysis was conducted in an academically-affiliated primary care teaching practice staffed mainly by general internists, some of whom were residents. However, we did not find any major diagnostic or prescribing differences between residents and non-residents. The presence of residents, researchers interested in judicious antibiotic use, and antibiotic-related quality improvement activities over the years may account for the relatively low antibiotic prescribing rate. Second, the analysis was dependent upon clinicians’ diagnoses and the accuracy and completeness of clinician documentation. However, as we were interested in clinician thinking about patients’ presentation and illness, the visit note provided insight into clinicians’ considerations. Third, our assignment of the most antibiotic-appropriate diagnosis as the principal diagnosis is forgiving to the treating clinicians and partially accounts for the very low apparent antibiotic prescribing rate for non-antibiotic-appropriate diagnoses. Fourth, expression of diagnostic uncertainty was varied and its interpretation somewhat subjective. Fifth, when assessing follow-up visits, we were limited to documentation within our healthcare system.

Despite these limitations, our analysis shows that acute cough visits are more complicated than guidelines and performance measures presume. Even in a practice in which the antibiotic prescribing rate is much lower than the national average, clinicians gave patients more than one diagnosis in nearly half of visits and explicitly expressed uncertainty in 16% of visits. Both diagnosis number and explicit uncertainty were positively associated with antibiotic prescribing. Such complexity and uncertainty is not reflected in guidelines or performance measures.

In addition to not capturing the complexity of actual clinical care – or perhaps because of it – the HEDIS acute bronchitis measure may cause unintended consequences. Roth and colleagues found that implementation of and improvement on the HEDIS measure led to a marked increase in a previously unused diagnosis code, which had a higher antibiotic prescribing rate [12]. If the goal of interventions is to decrease antibiotic prescribing for acute cough/acute bronchitis, our study suggests measures that include a broad range of diagnoses are necessary: acute bronchitis accounted for only 9% of acute cough visits and for only 6% of antibiotic prescribing for patients with acute cough. Likewise, guidelines may oversimplify the care of an acutely coughing adult into an idealized case of acute bronchitis, when, in fact, real patients are much more complicated [1,2].

## Conclusions

In conclusion, we found that 46% of acute cough visits had more than one cough-related diagnosis and clinicians expressed diagnostic uncertainty in 16% of visits. Antibiotic prescribing was associated with multiple diagnoses and diagnostic uncertainty. Revised guidelines that focus on the complaint of cough rather than on the diagnosis of “acute bronchitis”, and associated performance measures that include a broader range and number of diagnoses may more accurately reflect the complexity of the treatment of patients with acute cough.

Beyond use of broader measures and more realistic guidelines, we also need better interventions to improve care of patients with acute cough/acute bronchitis [14]. Most interventions for acute cough/acute bronchitis result in only a 10% absolute decrease in the antibiotic prescribing rate [15]. Some interventions have used decision support to address diagnostic decision-making [16,17], but efforts to markedly reduce antibiotic prescribing for acute cough likely need to directly address the diagnostic complexity and uncertainty faced by clinicians.

## Abbreviations

EHR: Electronic health record; HEDIS: The healthcare effectiveness data and information set; URI: Upper respiratory infection.

## Competing interests

The authors have no financial or non-financial competing interests related to this manuscript. No sponsor had a role in the design or conduct of the study; collection, management, analysis, or interpretation of the data; and preparation, review, or approval of the manuscript.

## Authors’ contributions

JAL conceived of the study and oversaw its implementation with ACB. LEW and PPD contributed to the data extraction, cleaning, and analysis. LEW and JAL participated in the statistical analysis. LEW drafted the manuscript and all authors contributed to the writing, editing, and final approval of this manuscript. All authors had full access to all the data in the study and take responsibility for the integrity of the data and the accuracy of the data analysis.

## Pre-publication history

The pre-publication history for this paper can be accessed here:

http://www.biomedcentral.com/1471-2296/14/120/prepub
